# Influence of dietary pattern on anti-tuberculosis treatment outcomes in persons with dysglycemia: a Peruvian prospective cohort study

**DOI:** 10.3389/fnut.2023.1254983

**Published:** 2023-12-18

**Authors:** María B. Arriaga, Mariana Araújo-Pereira, Vanessa M. B. Andrade, Artur T. L. Queiroz, Catarina D. Fernandes, Caio Sales, Juan Gonzalo Aliaga, Rupak Shivakoti, Leonid Lecca, Roger I. Calderon, Bruno B. Andrade

**Affiliations:** ^1^Division of Infectious Diseases, Department of Medicine, Vanderbilt University School of Medicine, Nashville, Tennessee, United States; ^2^Multinational Organization Network Sponsoring Translational and Epidemiological Research (MONSTER) Initiative, Salvador, Brazil; ^3^Laboratório de Pesquisa Clínica e Translacional, Instituto Gonçalo Moniz, Fundação Oswaldo Cruz, Salvador, Brazil; ^4^Instituto de Pesquisa Clínica e Translacional, Centro Universitário Faculdade de Tecnologia e Ciências, Salvador, Brazil; ^5^Center of Data and Knowledge Integration for Health (CIDACS), Instituto Gonçalo Moniz, Fundação Oswaldo Cruz, Salvador, Brazil; ^6^Curso de Medicina, Universidade Salvador (UNIFACS), Salvador, Brazil; ^7^Universidade Virtual do Estado de São Paulo. Cidade Universitária. Butantã, São Paulo, Brazil; ^8^Department of Epidemiology, Columbia University Mailman School of Public Health, New York, United States; ^9^Socios En Salud Sucursal Peru (Partners in Health), Lima, Peru; ^10^Department of Global Health and Social Medicine, Harvard Medical School, Boston, United States; ^11^Grupo de Investigación en Bioquímica y Biología Sintética, Universidad Nacional Federico Villarreal, Lima, Peru; ^12^Faculdade de Medicina, Universidade Federal da Bahia, Salvador, Brazil; ^13^Bahiana School of Medicine and Public Health, Bahia Foundation for the Development of Sciences, Salvador, Brazil

**Keywords:** tuberculosis, dysglycemia, dietary pattern, food group, systems nutrology, Peru, dietary intake, nutrition

## Abstract

**Introduction:**

Dietary patterns (DPs) are associated with overall nutritional status and may alter the clinical prognosis of tuberculosis. This interaction can be further intricated by dysglycemia (i.e., diabetes or prediabetes). Here, we identified DPs that are more common with tuberculosis–dysglycemia and depicted their association with tuberculosis treatment outcomes.

**Methods:**

A prospective cohort study of persons with tuberculosis and their contacts was conducted in Peru. A food frequency questionnaire and a multidimensional systems biology-based analytical approach were employed to identify DPs associated with these clinical groups. Potential independent associations between clinical features and DPs were analyzed.

**Results:**

Three major DPs were identified. TB–dysglycemia cases more often had a high intake of carbohydrates (DP1). Furthermore, DP1 was found to be associated with an increased risk of unfavorable TB outcomes independent of other factors, including dysglycemia.

**Conclusion:**

Our findings suggest that the evaluation of nutritional status through DPs in comorbidities such as dysglycemia is a fundamental action to predict TB treatment outcomes. The mechanisms underlying the association between high intake of carbohydrates, dysglycemia, and unfavorable tuberculosis treatment outcomes warrant further investigation.

## 1 Introduction

In 2014, the “END TB” Strategy of the World Health Organization (WHO) ambitiously proposed “ending the global tuberculosis (TB) epidemic” by 2035 (1). However, TB continues to be one of the leading causes of mortality worldwide, with more than 1.6 million deaths per year (2). Multiple studies have described various factors associated with the risk of developing TB, including HIV infection (3), smoking (4), alcohol abuse (5), diabetes mellitus (DM) (6), and malnutrition (7), among others.

In the past, we and others have described the association between TB and dysglycemia (i.e., DM and prediabetes) comorbidity with the clinical presentation (8), a greater number and variety of lung lesions (9), risk of treatment failure (10), and extended time to achieve bacteriological clearance in sputum (11). On the other hand, malnutrition heightens the likelihood of TB infection progressing to active disease (12).

Both dysglycemia and malnutrition can increase the incidence of TB (13, 14) and the risk of unsuccessful anti-TB treatment outcomes, such as failure, recurrence, and death (6, 14, 15). Peru, one of the South American countries most affected by TB, had approximately 28,892 new cases in 2019, of which 9.7% presented DM (16). Peruvian people with TB-DM comorbidity had a higher mortality rate (7.2%) between 2016 and 2018 than normoglycemic people (5.4%) in the same period (16). Currently, 2.7 million people are in a situation of malnutrition (17), and worryingly, in Peru, in 2020 the rates of failure (0.6%) and mortality during treatment (7.3%) were higher than in previous years (18).

Several studies have separately assessed the relationship of dietary intake with the risk of TB disease (19) and DM (20). Furthermore, other studies have reported the variation of the body mass index (BMI) among people with TB, before, during, and at the end of TB treatment (21), and underweight as a high-risk factor for TB-specific and non-TB-specific mortality during anti-TB treatment (ATT) (22). Nevertheless, nutrition studies have focused solely on specific foods or nutrients without considering the overall profile of food consumption. In this context, new approaches using dietary patterns, defined as a set of foods and the frequency, variety, and quantity consumed by individuals and populations (23), would make it possible to evaluate the diet from a global point of view and better explore the nutritional status of populations as complex as the TB–dysglycemia syndemic and its relationship with anti-TB treatment outcomes.

In a previous study, we used *big data* and artificial intelligence analysis tools to develop the concept of *systems nutrology* to identify dietary patterns of populations based on unsupervised statistical analysis (24, 25). The present study used our *systems nutrology* approach to characterize dietary patterns in a cohort of people with pulmonary TB and dysglycemia enrolled in Peru and to identify their association with anti-TB treatment outcomes.

## 2 Materials and methods

### 2.1 Study design

This study is a cross-sectional sub-study from a parent prospective cohort study of pulmonary TB patients and their household contacts, conducted between February and November 2017 in Lima, Peru, which was aimed at determining the prevalence of DM and prediabetes (preDM) in persons with TB. The study was carried out in the Public Hospital Sergio Bernales and outpatient health centers of the Carabayllo and Comas districts. People with pulmonary TB ≥18 years of age diagnosed by the Peruvian National TB Program who were not receiving ATT or had started it within a period of no more than 5 days were included. Exclusion criteria were patients or contacts diagnosed with HIV, pregnant people, those who did not live permanently in the jurisdiction area of the study, and patients who had infection or disease due to non-tuberculous mycobacteria.

The follow-up of TB cases and their household contacts was carried out up to 6–12 months after enrollment through three visits (baseline, month 2, and month 6). In each visit, clinical and epidemiological information (e.g., age, sex, and prior TB, among others) was collected. Additionally, specimens such as sputum and blood were also collected for analysis of hemoglobin (Hb), glycated hemoglobin (HbA1c), the oral glucose tolerance test (OGTT), and fasting plasma glucose (FPG) in all patients. The recruitment details and laboratory and field procedures are shown in the Supplementary Figure 1. More information can be found in a previous study with the same cohort (26). In this study, participants with TB-multi drug resistance (MDR) were excluded, and only data collected from the TB cases and their contacts at baseline were analyzed (before the TB cases initiated anti-TB treatment).

### 2.2 Sample size and study power

The previous prospective study was expected to enroll 130 individuals with TB and 130 contacts, considering 80% of power to a difference in DM prevalence of 10% between the study groups and a significance level of *α* = 0.05. In this study, 136TB cases and 133 household contacts were included, and a power of 81.9% was obtained (significance level of *α* = 0.05).

### 2.3 Definitions

TB cases were diagnosed in the health centers, according to the definitions of the Peruvian National TB Program (27), by one or more of the following criteria: (a) clinical factors (presumptive diagnosis), (b) bacteriology (sputum smear positive) or positive culture (solid or liquid), (c) GeneXpert MTB RIF, and (d) chest radiography. Contacts of TB cases were defined as a person aged 12 years or older, without TB diagnosis, who at least shared the household where they slept or ate their meals.

DM was defined in agreement with the American Diabetes Association (ADA) guidelines (28) as 2 h OGTT≥200 mg/dL, HbA1c ≥6.5%, or FPG ≥126 mg/dL. PreDM was also defined in agreement with the ADA guidelines as 2 h glucose 140 a 199 mg/dL, HbA1c 5.7–6.4%, or FPG 100–125 mg/dL. Dysglycemia was defined as DM or PreDM. In this study, participants were classified into dysglycemia, TB, TB–dysglycemia, and the control group, comprising non-TB/non-dysglycemia people.

Anemia was identified following the WHO criteria as Hb levels below 12 g/dL and 13 g/dL for female and male patients, respectively.

Finally, TB treatment outcomes such as recurrence, treatment modification, treatment failure, and death (during treatment) were classified as unfavorable outcomes. Cure and complete treatment were defined as favorable outcomes. All definitions were established according to the Peruvian National TB Program (27) and are described in the Supplementary Table 1.

### 2.4 Food consumption data collection

The food intake was obtained according to a food frequency questionnaire (FFQ), with 94 food items. The FFQ was specifically developed for this study to investigate and delineate the dietary patterns of people with TB and their household contacts with and without dysglycemia among the Peruvian population and has thus not been validated in previous studies. In addition, the data retrieving and processing of variables were performed following standardized steps from a previous study of our group (24, 25).

The FFQ was applied to TB cases and their household contacts at the enrollment visit (before the patient initiated anti-TB treatment). The consultations on habitual diet referred to the consumption of food outside and inside the home in the last 6 months. The food frequency choices had the following response options: Never / rare; 1 to 3 times a month; 1 time per week; 2 to 4 times a week; and ≥ 4 times a week. In addition, the number of times these foods were consumed was investigated. After data collection, using food composition and nutrition tables, we standardized the amounts consumed of each food and/or preparation, referred to units of weight (g) and/or volume (mL), which were used for calculating the daily consumption of the food recorded in the FFQ. Industrialized foods and / or preparations that were not included in the tables were searched through the Internet directly on the manufacturer’s website or through recipes. Thus, it was possible to obtain an approximation of the total daily food consumption in grams through calculations based on weekly and monthly consumption.

The average daily consumption in grams of these food items was provided by the self-reports of the participants. For the statistical analysis, the food items that composed the FFQ were categorized into 10 food groups according to the similarity in nutritional composition and food habits of the Peruvian population: Sugar and sweets, sweetened beverages, tubers, fast food, oils, milk and dairy, fruits and vegetables, rice and cereals, meat, and legumes. The approach used for creating these groups is described in Supplementary Table 2.

### 2.5 Statistical data analysis

To analyze the potential association between food consumption profiles and anthropometric and dysglycemia status, we used an analytical approach denominated ‘*systems nutrology’*, created and detailed by our group in the article of Andrade et al., based on the ecological analysis approach (24, 25).

To evaluate the overall consumption profile of the different food groups, we transformed data on the total consumption of each food group (in grams) into the abundance of consumption relative to total diet, or relative abundance (24, 25). After defining the proportion of consumption of each food group in the total ingestion, we performed an unsupervised hierarchical cluster analysis to define the dietary patterns (Ward’s method). The different clusters, named dietary patterns (DPs), were defined by the overall similarity of food consumption, represented using dendrograms denoting the Euclidean distances. We also used bubble plots to illustrate the representativeness of consumption of each food group in each one of the DPs calculated. Pie charts were used to illustrate the frequency of individuals grouped by clinical group, sex, and anemia status in each DP.

Characteristics of the study participants were presented as median and interquartile ranges (IQR) for continuous variables or frequency (%) for categorical variables. The Gaussian distribution of continuous variables was assessed using the Kolmogorov–Smirnov test (no variable was normally distributed) and compared using the Mann–Whitney *U* test (between two groups) or Kruskal–Wallis test (between more than two groups) with Dunn’s and Tukey’s multiple comparisons. Categorical variables were compared using the Fisher’s exact test (2 × 2 comparisons) or Pearson’s chi-square test (more than two groups). For comparisons of abundance of food consumption, one-way ANOVA was used.

Correlations between total intake in grams of each food group and clinical variables associated with glucose levels (HbA1c and FPG) were evaluated by the Spearman rank test. We created correlation matrices stratifying the participants according to TB status.

To test the association between the unfavorable ATT outcome and DP identified in the hierarchical cluster analysis, we performed a binary logistic regression analysis. The selection of variables was based on Lasso regression. Seventy percent of the cases were randomly selected as the training set, and the rest of the cases were included in the validation set for the internal validation of the model. The DP2 was used as the baseline for the estimation of odds of consumption of the other patterns, given that most patients in this cluster were non-TB/non-dysglycemia. The results were presented in the form of adjusted odds ratio (aOR) and 95% confidence intervals (CIs).

All the analyses and data visualizations were pre-specified. Two-sided *p* value < 0.05 after adjustment for multiple comparisons (Bonferroni’s method) was considered statistically significant. Statistical analyses were performed using R 3.4.2 (R Foundation) and SPSS 24.0 (IBM statistics). The R packages used were ComplexHeatmap v2.2.0, Hmisc v4.4.1, and ggplot2 v3.3.2.

## 3 Results

### 3.1 Characteristics of the study participants according to TB and dysglycemia status

Our cohort was composed of 269 individuals from Lima, Peru. Among the participants in the study, 85 (31.59%) were considered non-TB/non-dysglycemia, 48 (17.84%) had dysglycemia, 75 (27.88%) had TB, and 61 (22.67%) had TB and dysglycemia. The median age was highest for people with dysglycemia at 50.6 years (IQR: 40.2–61.0), while TB patients had the lowest median age at 25.8 years (IQR: 21.3–31.0, *p* < 0.001). In this study, there were 137 male and 132 female participants; however, the frequencies of male participants in the investigated groups (dysglycemia, TB and TB–dysglycemia) were higher than that of female participants. In the control group, with non-TB/non-dysglycemia participants, this proportion was inverse (*p* = 0.002) (Table 1).

**Table 1:** Characteristics and relative abundance of food group consumption of the study population according to TB and dysglycemia status.

Parameter	non-TB/non-dysglycemia	Dysglycemia	TB	TB–dysglycemia	value of *p*
	*n* = 85	*n* = 48	*n* = 75	*n* = 61	
Age (years), median (IQR)	31.0 (19.8–44.2)	50.6 (40.2–61.0)	25.8 (21.3–31.0)	43.3 (29.7–53.9)	**<0.001**
Male, n. (%)	29 (34.1)	25 (52.1)	47 (62.7)	36 (59.0)	**0.002**
Illiteracy, n. (%)	18 (21.2)	7 (14.6)	28 (37.3)	6 (9.84)	**0.001**
Prior TB, n. (%)	9 (10.6)	5 (10.4)	10 (13.3)	13 (21.3)	0.249
Smoking, n. (%)	12 (14.1)	7 (14.6)	15 (20.0)	14 (23.3)	0.455
Passive smoking, n. (%)	11 (12.9)	8 (16.7)	5 (6.67)	6 (10.0)	0.342
Cannabis use, n. (%)	0 (0.00)	2 (4.17)	13 (17.3)	8 (13.3)	**<0.001**
Illicit drug use, n. (%)	0 (0.00)	1 (2.08)	8 (10.7)	9 (15.0)	**<0.001**
Alcohol use, n. (%)	24 (28.2)	24 (50.0)	37 (49.3)	33 (55.0)	**0.004**
Anemia, n. (%)	32 (37.6)	10 (20.8)	49 (66.2)	50 (82.0)	**<0.001**
BMI (kg/m^2^), median (IQR)	26.1 (22.9–29.4)	29.6 (27.9–33.3)	22.3 (20.4–25.2)	23.0 (21.5–25.7)	**<0.001**
Waist (cm), median (IQR)	88.0 (79.0–94.0)	98.0 (93.8–106)	80.0 (74.0–86.0)	84.0 (78.0–89.0)	**<0.001**
Hb (g/dL), median (IQR)	13.2 (12.2–14.2)	13.6 (12.8–14.2)	12.6 (11.2–13.4)	11.4 (10.2–12.6)	**<0.001**
FPG (g/dL), median (IQR)	90.6 (87.5–94.1)	106 (102–114)	89.9 (85.8–94.6)	104 (99.8–136)	**<0.001**
HbA1c (%), median (IQR)	4.90 (4.60–5.20)	5.30 (4.90–5.73)	5.00 (4.70–5.20)	5.60 (5.10–6.75)	**<0.001**
Food group (g/day)					
Total consumption, median (IQR)	981 (885–1,095)	1,232 (1,160–1,350)	1,046 (963–1,225)	1,616 (1,459–1,733)	**<0.001**
Rice and cereals, median (IQR)	490 (474–506)	574 (567–594)	557 (430–565)	645 (610–651)	**<0.001**
Tubers, median (IQR)	187 (175–191)	237 (233–247)	233 (163–233)	275 (257–278)	**<0.001**
Milk and dairy, median (IQR)	69.3 (34.7–107)	139 (69.3–213)	34.7 (34.7–61.3)	76.7 (43–139)	**<0.001**
Fruits and vegetables, median (IQR)	52 (34.7–78)	34.7 (26.0–65)	8.7 (8.7–13)	13 (13–21)	**<0.001**
Legumes, median (IQR)	45.5 (31.5–49)	14.0 (14.0–17.5)	17.5 (10.5–21)	17.5 (10.5–21)	**<0.001**
Meat, median (IQR)	50.3 (35.5–67.3)	56.6 (40.8–71.5)	33 (25–40)	42.2 (29.7–53.7)	**<0.001**
Fast food, median (IQR)	60.7 (28.3–74.7)	55.5 (51.3–108)	53.7 (18.7–74.7)	213 (112–427)	**<0.001**
Sweetened beverages, median (IQR)	33.3 (33.3–83.3)	33.3 (4.2–83.3)	33.3 (8.33–83.3)	233 (133–267)	**<0.001**
Sugar and sweets, median (IQR)	20 (20–20)	20 (10–25)	18 (10–20)	40 (20–45)	**<0.001**
Oils, median (IQR)	5.3 (2.67–8)	10.7 (7.3–21.3)	2. 7 (2.7–5.3)	10.7 (6.7–21.3)	**<0.001**

The data presented in the continuous variables (represented median and interquartile range [IQR]) between clinical groups were compared using the Kruskal–Wallis test with Dunn’s multiple comparisons post-test. Qualitative variables were represented by number and frequency (%) and compared using Pearson’s chi-square test. Value of *p*s were adjusted for multiple measurements using the Holm–Bonferroni method.

TB, tuberculosis; BMI, body mass index; Hb, hemoglobin; OGTT, oral glucose tolerance test; FPG, fasting plasma glucose; HbA1c, glycated hemoglobin. Bold values indicates statistical significance.

The TB–dysglycemia group presented the lowest frequency of illiteracy (Control: 21.2%; Dysg: 16.6%; TB: 62.4%; TBdysg: 9.84%, *p* = 0.001) and higher frequencies of illicit drug use (Control: 0.0%; Dysg: 2.1%; TB: 10.7%; TBdysg: 15.0%, *p* < 0.001), alcohol abuse (Control: 28.2%; Dysg: 50%; TB: 49.3%; TBdysg: 55%, *p* = 0.004), and anemia (Control: 37.6%; Dysg: 20.8%; TB: 66.2%; TBdysg: 82.0%, *p* < 0.001), as well the lowest level of hemoglobin [Control: 13.2 (12.2–14.2); Dysg: 13.6 (12.8–14.2); TB: 12.6 (11.2–13.4); TBdysg: 11.4 (10.2–12.6), *p* < 0.001] (Table 1). TB cases presented lower values of BMI [Control: 26.1 (22.9–29.4); Dysg: 29.6 (27.9–33.3); TB: 22.3 (20.4–25.2); TBdysg: 23.0 (21.5–25.7), *p* < 0.001]; and waist circumference [Control: 88.0 (79.0–94.0); Dysg: 98.0 (93.8–106); TB: 80.0 (74.0–86.0); TBdysg: 84.0 (21.5–25.7), *p* < 0.001]. The overall comparisons of clinical data according to TB and dysglycemia status are detailed in Table 1.

### 3.2 Food intake between clinical groups

In the comparisons of food group intake (grams per day), we observed statistical differences for all the food groups between the clinical groups, with *p* < 0.001 (Table 2). Non-TB/non-dysglycemia individuals had lower intakes of tubers, rice, and cereals and higher intakes of legumes, fruits, and vegetables compared to the other groups. The dysglycemia group had lower intakes of legumes and higher intakes of meat and milk and dairy. The TB participants presented the lowest intakes of milk and dairy, fruits and vegetables, meat, fast food, sugar and sweets, and oils. Finally, the TB–dysglycemia group presented the highest total consumption, with higher intakes of rice and cereals, tubers, fast food, sweetened beverages, sugar and sweets, and oils. The values for each food group according to TB and dysglycemia status are described in Table 1.

**Table 2:** Characteristics and relative abundance of food group consumption of the study population according to dietary patterns.

Parameter	Dietary pattern 1	Dietary pattern 2	Dietary pattern 3	*p*-value
	*n* = 58	*n* = 70	*n* = 141	
Clinical groups, n. (%)				**<0.001**
Non-TB/non-dysglycemia	0 (0)	61 (87.1)	24 (17.0)	
Dysglycemia	3 (5.2)	9 (12.9)	36 (25.5)	
TB	2 (3.4)	0 (0.00)	72 (51.1)	
TB–dysglycemia	53 (91.4)	0 (0.00)	9 (6.4)	
Age (years), median (IQR)	44.6 (28.4–54)	36.5 (24–51.2)	28.9 (22.5–43.8)	**<0.001**
Male, n. (%)	36 (62.1)	28 (40)	73 (51.8)	**0.002**
Illiteracy, n. (%)	6 (10.3)	13 (18.6)	40 (28.4)	**0.002**
Prior TB, n. (%)	12 (20.7)	8 (11.4)	17 (12.1)	0.571
Smoking, n. (%)	11 (19.3)	10 (14.3)	27 (19.1)	0.411
Passive smoking, n. (%)	6 (10.5)	10 (14.3)	15 (9.9)	0.557
Cannabis use, n. (%)	7 (12.3)	1 (1.4)	10 (10.6)	**0.008**
Illicit drug use, n. (%)	8 (14)	0 (0.00)	10 (7.1)	**0.005**
Alcohol use, n. (%)	30 (52.6)	26 (37.1)	62 (44)	**0.005**
Anemia, n. (%)	46 (79.3)	23 (32.9)	72 (51.4)	**<0.001**
BMI (kg/m^2^), median (IQR)	23.8 (21.9–26.5)	27.7 (26–30.3)	23.5 (20.9–27.2)	**<0.001**
Waist (cm), median (IQR)	86.0 (78–91)	92 (87–100)	83 (76–91)	**<0.001**
Hb (g/dL), median (IQR)	11.7 (10.3–13.1)	13.3 (12.7–14.4)	12.8 (11.7–13.9)	**0.014**
FPG (g/dL), median (IQR)	104.2 (99.3–150.4)	92.2 (89–96.3)	92.5 (87.5–100.2)	**<0.001**
HbA1c (%), median (IQR)	5.6 (5.1–6.8)	5 (4.6–5.3)	5 (4.7–5.3)	**<0.001**
Relative abundance by food group (%)				
Rice and cereals	139 (69.3–213)	34.7 (34.7–61.3)	18.0 (12.7–213)	**<0.001**
Tubers	0.52 (0.48–0.56)	0.47 (0.42–0.49)	0.41 (0.37–0.45)	**<0.001**
Milk and dairy	0.03 (0.01–0.04)	0.02 (0.03–0.08)	0.16 (0.01–0.17)	**<0.001**
Fruits and vegetables	0.11 (0.09–0.23)	0.28 (0.16–0.29)	0.14 (0.13–0.19)	**<0.001**
Legumes	0.02 (0.01–0.02)	0.10 (0.03–0.05)	0.03 (0.01–0.05)	**<0.001**
Meat	0.03 (0.01–0.04)	0.06 (0.01–0.08)	0.04 (0.02–0.06)	**<0.001**
Fast food	0.22 (0.03–0.29)	0.09 (0.03–0.11)	0.05 (0.02–0.21)	**<0.001**
Sweetened beverages	0.13 (0.01–0.17)	0.03 (0.01–0.08)	0.04 (0.02–0.15)	**<0.001**
Sugar and sweets	0.32 (0.01–0.02)	0.05 (0.01–0.07)	0.04 (0.01–0.08)	**<0.001**
Oils	0.04 (0.00–0.06)	0.01 (0.00–0.01)	0.01 (0.00–0.01)	**<0.001**

The data presented in the continuous variables (represented median and interquartile range [IQR]) between food groups were compared using the Kruskal–Wallis test with Dunn’s multiple comparisons post-test. *P*-values were adjusted for multiple measurements using the Holm–Bonferroni method. TB, tuberculosis; BMI, body mass index; Hb, hemoglobin; HbA1c, glycated hemoglobin. Bold values indicates statistical significance.

### 3.3 Dietary patterns associated with TB–dysglycemia

**Figure 1:**
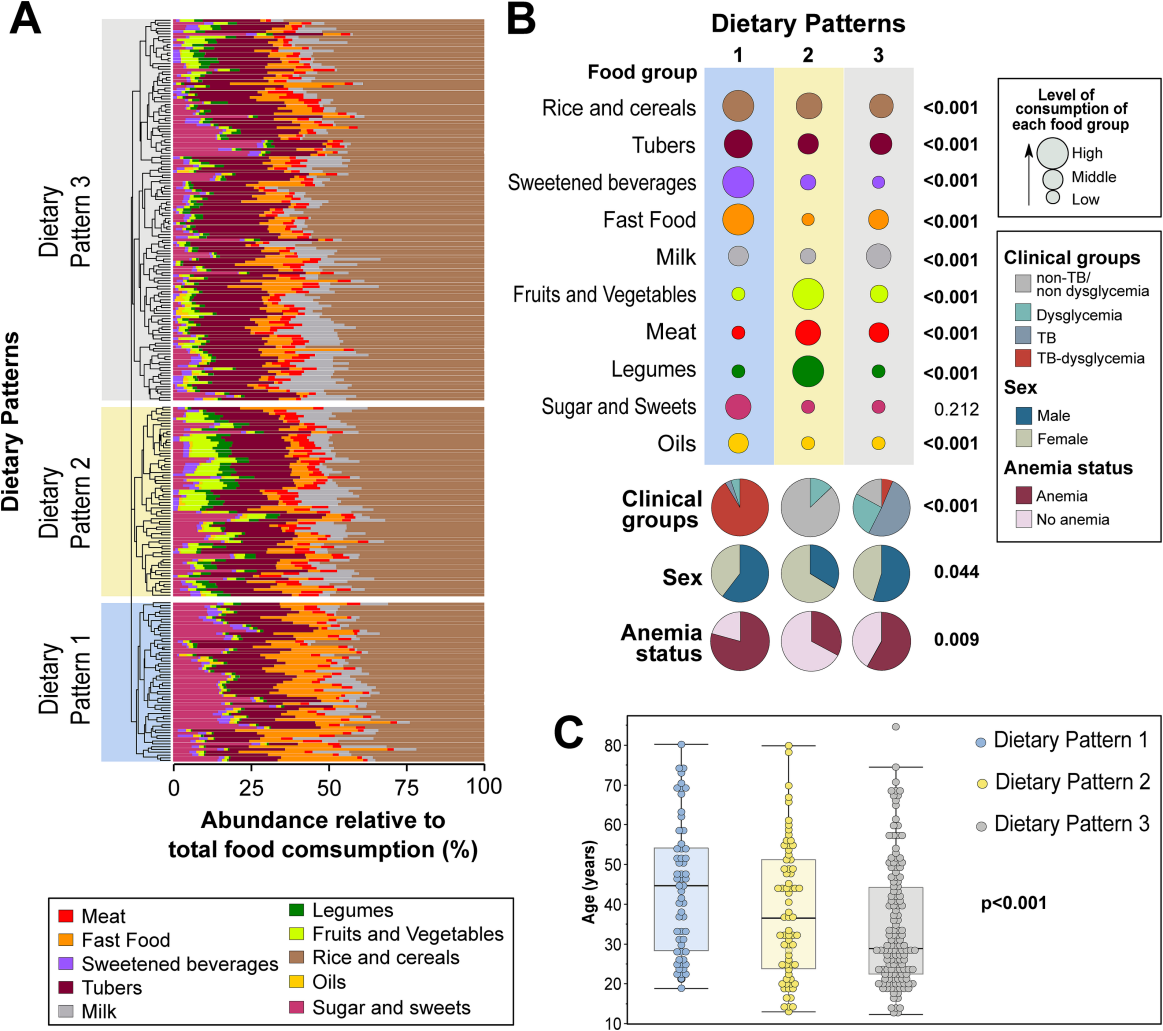
Dietary patterns of food groups using hierarchical clustering. The abundance of consumption of the indicated food groups in the diet was calculated for each one, as described in the Methods. **(A)** Hierarchical cluster analysis using Ward’s unsupervised method. The dendrograms represent the Euclidean distance and were used to identify different consumption profiles. Using this approach, it was possible to identify three major dietary patterns. **(B)** Upper panel shows the abundance of consumption, stratified into high, middle, and low, of each food group within each dietary pattern identified in the hierarchical cluster analysis. Lower panel shows frequencies of the individuals in each dietary pattern stratified by clinical groups, sex, and anemia. **(C)** Scatters plots display the age distribution in each dietary pattern. To calculate the value of *p*s, the average relative abundance of consumption of each food group was compared between the different dietary patterns using two-tailed one-way ANOVA. Proportions of clinical groups, sex, and anemia were compared between the different dietary patterns using the Pearson’s chi-square test. The Kruskal–Wallis test was used to compare the age distribution between dietary patterns. All value of *p*s are indicated. The values of this analysis are detailed in Table 2. TB, tuberculosis. Bold values indicates statistical significance.

An unsupervised hierarchical cluster analysis (Ward’s method) of the abundance of food consumption (which infers the representativeness of the intake of each food group in the overall diet) was used to identify DPs of the consumption of distinct food groups, independent of the study group (non-TB/non-dysglycemia, dysglycemia, TB, and TB–dysglycemia). It was possible to identify three major DPs: DP1 (*n* = 58), DP2 (*n* = 70), and DP3 (*n* = 141) (Figure 1A and Table 2). All TB cases (with and without dysglycemia) were distributed in DP1 and DP3, while no TB patient was grouped in DP2. DP1 was represented mostly by TB–dysglycemia cases (91.4%), while in DP2, there were more frequent control individuals (87.1%). Meanwhile, DP3 was composed mainly of TB cases (51.1%) (Figure 1B and Table 2). While analyzing the DP according to the relative abundance of the food groups consumed, it was possible to observe that the rice and cereals group was the most representative food group in all the three clusters, followed by tubers (Figure 1A). However, in comparing the groups with each other, we observed that DP1 exhibited the highest intakes of these food groups, including sweetened beverages and fast food, and the highest frequency of persons of male sex and with anemia (Figure 1B and Table 2). In contrast, DP2 presented the highest frequency of female participants (*p* < 0.001) and displayed higher intakes of legumes, fruits, and vegetables, while DP3 presented the highest intakes of milk (Figure 1B and Table 2). Furthermore, DP1 showed a higher median age than DP2 and DP3 (*p* < 0.001) (Figure 1C and Table 2). Additional analyses testing associations between the DPs and the clinical groups are detailed in Table 2.

In general, DP1 was predominantly composed of individuals who related the use of cannabis (12.3%, *p* = 0.008) and illicit drugs (14%). In addition, this DP presented the highest values of BMI (23.8 kg/m2, IQR: 21.9–26.5, *p* < 0.001), FPG (104.2 g/dL, IQR: 99.3–150.4, *p* < 0.001), and HbA1C (5.6%, IQR: 5.1–6.8, *p* < 0.001), in contrast with other DPs. In contrast, DP1 presented the lowest Hb levels (11.7 g/dL, IQR: 10.3–13.1, *p* < 0.001) in comparison with other groups, as shown in Table 2. DP3 was composed mainly of individuals with the highest frequency of illiteracy (28.4%, *p* = 0.002) and lowest BMI values (23.5 kg/m^2^, IQR: 20.9–27.2, *p* < 0.001). Finally, DP2 was composed of a small frequency of male participants (40%, *p* = 0.002), without illicit drug or cannabis use reported, and the highest BMI (27.7, IQR: 26–30.3, *p* < 0.001), waist circumference (92 cm, IQR: 87–100, *p* < 0.001), and Hb levels (13.3, IQR: 12.7–14.4, *p* = 0.014) (Table 2).

**Figure 2:**
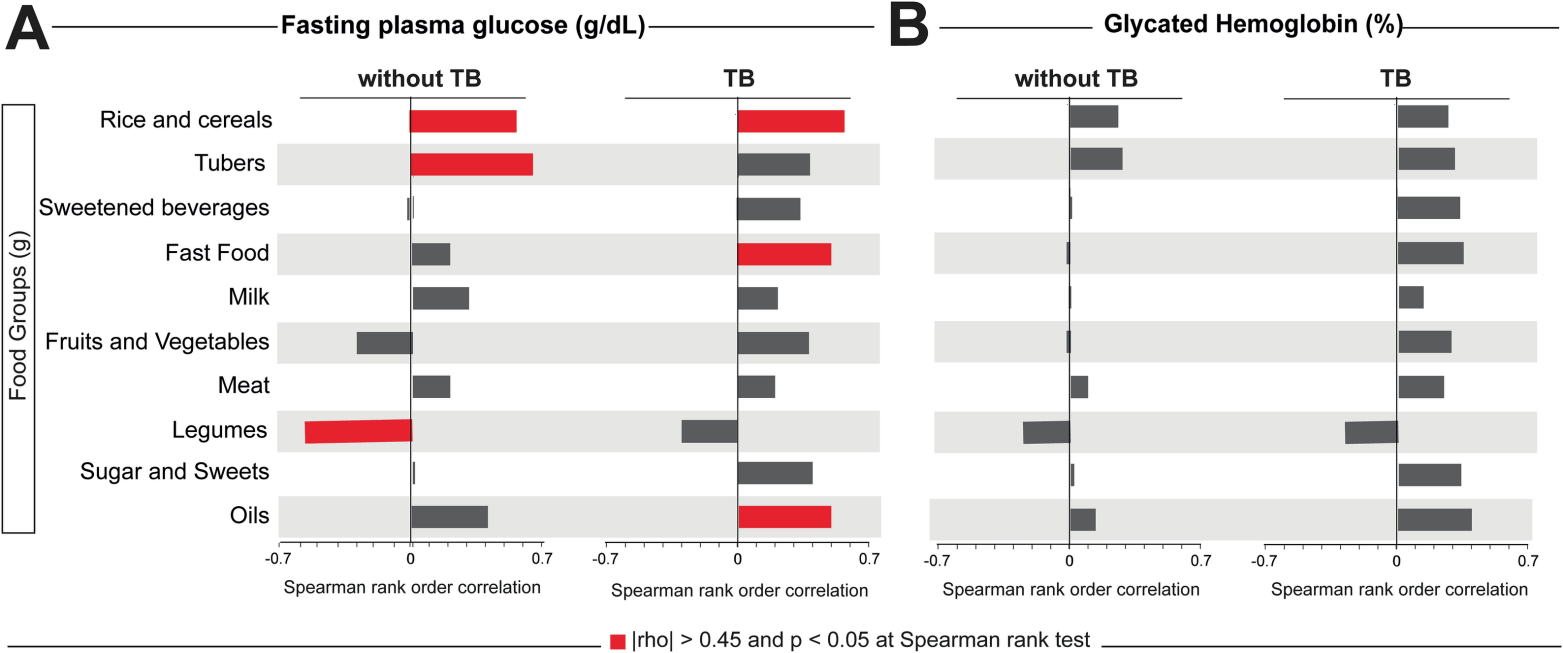
Correlations between food consumption and values of FPG and HbA1c. A Spearman correlation test was performed between fasting plasma glucose **(A)** or glycate hemoglobin **(B)** and the total consumption in grams of each food group. Significative correlations (|rho| > 0.45 and *p* < 0.05) for each glycemic test and group are highlighted in red bars. TB, tuberculosis.

To evaluate the influence of the intake of food groups on the glycemic status of the study participants (assessed here through FPG and HbA1c levels), we performed a Spearman correlation test between these two variables and each food group, stratifying the participants according to TB status. In our cohort, we observed that there were higher correlations between some food groups and FPG (Figure 2A), but no statistically significant correlation was observed with HbA1C frequency (Figure 2B). In individuals without TB, consumption of rice and cereals and tubers was positively correlated with FPG levels, while consumption of legumes was negatively correlated with this variable. In TB cases, besides the consumption of rice and cereals, fast food and oils were also positively correlated with FPG levels (Figure 2A).

### 3.4 Characteristics and food intake according to treatment outcome

When we classified the TB population according to treatment outcome, 28 individuals (23%) had an unfavorable outcome [treatment modification (*n* = 4), loss to follow-up (*n* = 5), failure (*n* = 8), and death (*n* = 11)], while 92 individuals (77%) completed treatment favorably (Supplementary Table 3). TB cases with unfavorable outcomes were characterized by elevated HbA1c levels (5.3% vs. 5.1%, *p* = 0.004) and FPG (100 g/dL vs. 93.5 g/dL, *p* = 0.003) compared to TB cases that had a favorable treatment outcome. Consequently, 85.7% of individuals with unfavorable outcomes had dysglycemia, whereas only 30.4% of individuals with a favorable outcome had this condition (*p* < 0.001) (Supplementary Table 3).

Regarding consumption according to food group, notably, participants who experienced unfavorable outcomes had, at baseline, higher consumption of rice and cereals, tubers, fast food, sweetened beverages, sugar and sweets, and oils. In contrast, individuals with a favorable outcome exhibited a lower intake of the aforementioned food groups (*p* < 0.05) (Supplementary Table 3).

### 3.5 Association between dietary patterns and TB treatment outcomes

**Figure 3:**
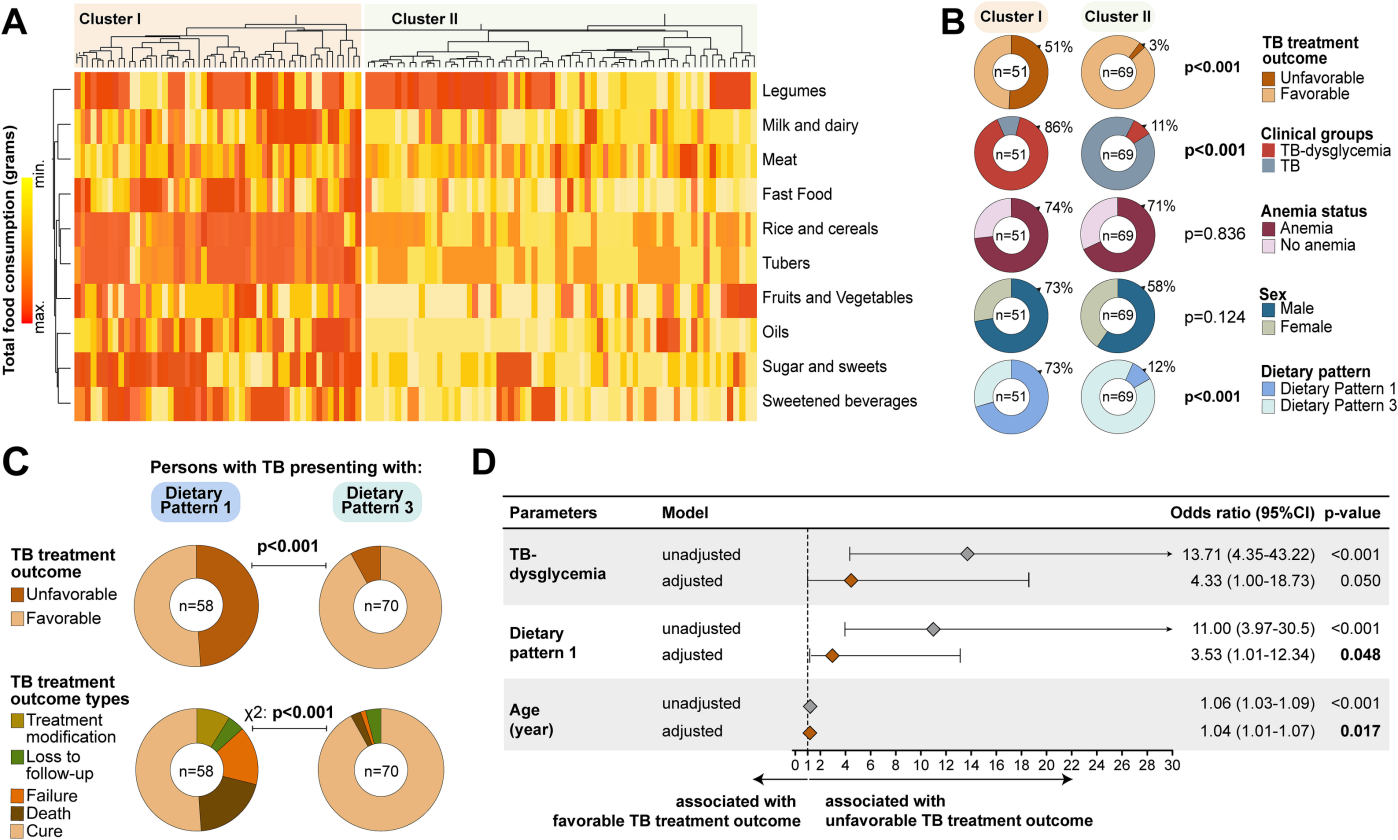
Dietary patterns associated with unfavorable TB treatment outcome. **(A)** Hierarchical cluster analysis employing Ward’s unsupervised method. In this approach, we included the TB treatment outcome variable, and it was possible to identify two major clusters. Details about Cluster 1 and Cluster II are described in Supplementary Table 4. **(B)** Panel shows frequencies individuals in each cluster stratified by TB treatment outcome, clinical groups, anemia status, sex, and dietary pattern. **(C)** Panel displays the distribution of TB treatment outcome through dietary patterns 1 and 3. **(D)** Logistic regression to test independent associations with the TB treatment outcome. Adjustment was performed for TB–dysglycemia, DP1, and age variables presented in the figure. Dietary pattern 3 was used as a reference to test associations between the variables and dietary pattern 1. Variables selection for logistic regression was performed using Lasso regression analysis (Supplementary Table 5). TB, tuberculosis; CI, confidence interval; BMI, body mass index. Bold values indicates statistical significance.

We performed a second hierarchical cluster analysis among only people with TB using food groups, clinical parameters, dietary patterns (DP1 and DP3, identified in this study subpopulation), and TB treatment outcomes (classified as unfavorable and favorable). This analysis resulted in two major clusters (Figure 3A). Cluster I displayed a profile characterized by significantly greater consumption of all food groups, except legumes (Figure 3A and Supplementary Table 4). Interestingly, in Cluster I, 51% of the study participants experienced an unfavorable outcome, and this percentage was substantially higher than the 4% detected in Cluster II (*p* < 0.001). Furthermore, Cluster I was mostly composed of people with TB–dysglycemia (86%, *p* < 0.001) and higher frequency of illicit drug use (19.6%, *p* = 0.041), medians of age (37.8 years), HbA1c (5%), and FPG (100.8 g/dL) compared to Cluster II. More details about Cluster I and Cluster II are presented in Supplementary Table 4. Notably, participants from Cluster I more frequently had DP1 (73%) than those in Cluster II (12%) (*p* < 0.001) (Figure 3B). Individuals who had DP1 more frequently experienced unfavorable TB treatment outcomes (49%) when compared with those who had DP3 (8%) (*p* < 0.001); death was the most frequent outcome in persons who had DP1 (Figure 3C).

Finally, to test for an independent association between the DPs identified and the clinical groups with a risk of developing an unfavorable TB treatment outcome, we performed a logistic regression analysis. Using this approach, we found that DP1 (aOR: 3.53, 95%CI: 1.01–12.34, *p* = 0.048) and age (aOR: 1.04, 95%CI: 1.01–1.07, *p* = 0.017) were associated with increased risk of unfavorable TB treatment outcomes, independently of TB–dysglycemia status (adjusted Odds Ratio [aOR]: 4.33, 95%CI: 1.00–18.73, *p* = 0.050) (Figure 3D).

## 4 Discussion

The association between nutrition and human health has been widely studied, and evidence suggests that dietary habits can impact the development of various human pathologies (29) such as dysglycemia and TB (13, 14). The emerging field of systems nutrology, which utilizes big data and *systems biology* algorithms, provides a new approach to understanding the interplay between nutrition and disease. In this study, we used this approach to identify dietary patterns in a cohort of Peruvian people with and without TB, stratified according to dysglycemia. Our findings revealed a distinct dietary pattern in the TB–dysglycemia group, which was associated with an increased risk of unfavorable anti-TB treatment outcomes. The initial analyses of our study demonstrated a high frequency of dysglycemia in the Peruvian population with TB, which is consistent with previous reports on DM prevalence in Peru (16) and in similar cohorts from low-and middle-income countries, such as South Africa, India, and Brazil (30).

The exact mechanisms underlying the association between dysglycemia and TB are not fully understood. Nevertheless, it is believed that dysglycemia may impair the ability to fight TB infection (6), while TB can exacerbate dysglycemia by causing stress hyperglycemia, which can influence the clinical manifestation and ATT response (31). It was also revealed that the TB–dysglycemia group more frequently reported anemia, which may be related to immunological deterioration (32), more advanced disease, and other comorbidities (33). Of note, tobacco, alcohol, and substance use were all more frequently observed in this group. Both conditions (i.e., dysglycemia and anemia) may be related with food consumption habits (34–36). However, to our knowledge, there have been no studies evaluating the association between these factors in TB patients. Nevertheless, a meta-analysis in a population non-TB showed that dietary patterns clustered according to food groups were associated with diabetes risk (37). Therefore, our study sheds light on this association, which may help in the clinical and nutritional management of TB and/or dysglycemic patients to improve their quality of life and prognosis.

The results of the hierarchical clustering analysis of the food consumption data revealed three distinct dietary patterns in the population, regardless of TB or dysglycemia. DP1 was predominantly composed of TB–dysglycemia patients (91%) and was characterized by a higher intake of sweetened beverages and fast food. In contrast, in DP2, most participants were those without TB and dysglycemia (87%), and the pattern was characterized by a relatively higher intake of fruits, vegetables, and legumes. Almost half of DP3 was composed of TB cases, and this pattern included people with higher milk consumption. The frequency of participants of each group in each DP agrees with the correlation analysis results, where we observed that the consumption of fast food was associated with higher FPG, while the consumption of legumes was associated with lower levels of FPG. Thus, our results confirm the association between food consumption habits and dysglycemia and agree with our initial hypothesis that there is a specific DP associated with TB–dysglycemia.

According to the Dietary Guidelines for Americans (2020–2025) (23), the DP1 found in our study is the opposite of the definition of a healthy DP, described as the habit of consuming foods and beverages rich in nutrients, including the consumption of vegetables, fruits, grains, dairy products (low-fat or fat-free, preferably), and protein foods and oils, plus the limited consumption of added sugars and saturated fats (23). Meanwhile, the DP2 described here fits the definition of a healthy DP. In parallel, DP3 seems to be in a position between the healthy and unhealthy DPs. Altogether, these results expose that TB patients have an unhealthy DP, and this is more pronounced in TB–dysglycemia patients. Whether the unhealthy DP influenced dysglycemia and/or TB infection, or the opposite, could not be evaluated in this study. However, these results can serve as an important guide to direct the nutritional management of these patients at the time of TB–dysglycemia diagnosis.

There are elements that influence the establishment of a DP (23, 24), such as personal preferences (e.g., the taste of food), cultural (e.g., the influence of family and friends), and traditional (e.g., the use of regional foods and the way of cooking) factors, as well as the accessibility and availability of food. The food groups of rice and cereals and tubers were the most representative in all three DPs identified in the study population. The DPs of Latin America countries have traditionally been marked by a strong presence of foods based on cereals, roots, and tubers. In Peru, estimates indicate that they provide half of the energy in the diet (38). Despite rice and cereals showing a positive correlation with FPG values, there are other food groups that are specifically associated with the increase of this marker in TB patients, such as fast food and oil consumption. Lima concentrates more than 75% of formal fast-food stalls in Peru, and it is estimated that there are more than 22,000 fast-food stalls in the country (39). The high consumption of carbohydrates, especially with high glycemic index as found in fast foods, is associated with cardiovascular diseases (40) and can be related with inflammation (41). In the context of TB, this exacerbated inflammation hypothetically associated with the distinct DPs can be associated with a heightened risk of unfavorable outcomes. Thus, the DP should be explored as a comorbidity.

In order to test the associations between the DPs and risk of unfavorable anti-TB treatment outcomes, we performed robust systems nutrology analysis. Among the study participants with TB, we found that DP1 was independently associated with unfavorable TB treatment outcomes. A previous study showed that an unhealthy DP is associated with all-cause mortality, cardiovascular disease, overweight, obesity, and DM, among others (42), due to micronutrient deficiencies leading to pathophysiological events that cause nutritional stress, which weakens and limits the immune response (6, 14, 15). This context could explain the association of DP1 and TB–dysglycemia with unfavorable TB treatment outcomes. In addition, an increase in age was also independently associated with an unsuccessful TB treatment, perhaps because aging is related to increased oxidation (43) and systemic inflammatory disturbance in the context of TB (43).

This study has some limitations. Socioeconomic data or physical activity were not collected in enrolled individuals to evaluate the relationship with DP. Food consumption information was self-reported, which could have induced a recall bias. The data were collected 5 years ago and only in the city of Lima (it was not a multi-site study), and we did not use a standard FFQ or extrapolate the study results with caution. These limitations indicate that the findings reported here should ideally be validated in other studies with distinct clinical and epidemiologic settings. Regardless of such limitations, our study contributes to the current knowledge in the field by providing strong novel evidence demonstrating the impact of dietary pattern on TB treatment outcomes, independent of the presence of dysglycemia.

The results presented here demonstrate the association between a dietary pattern characterized by the consumption of sweetened beverages, fast food, and milk and unfavorable TB treatment outcomes, independent of other confounding factors such as dysglycemia. Studying food consumption patterns is key to determining the available food system as a manifestation of the functioning of food and, in turn, determining the nutritional status and especially the health of the population.

## Data availability statement

The raw data supporting the conclusions of this article will be made available by the authors, without undue reservation.

## Ethics statement

The studies involving humans were approved by Institutional Committee of Ethics for Humans (CIEI, approval number: 158-22-16) of the Universidad Peruana Cayetano Heredia, with the authorization of National Institute of Health in Peru. The studies were conducted in accordance with the local legislation and institutional requirements. The participants provided their written informed consent to participate in this study.

## Author contributions

MA: Conceptualization, Data curation, Formal analysis, Investigation, Methodology, Software, Supervision, Validation, Visualization, Writing – original draft, Writing – review & editing. MA-P: Formal analysis, Writing – original draft. VA: Conceptualization, Formal analysis, Investigation, Methodology, Writing – original draft. AQ: Data curation, Formal analysis, Methodology, Writing – original draft. CF: Investigation, Methodology, Writing – original draft. CS: Investigation, Methodology, Writing – original draft. JA: Investigation, Methodology, Writing – original draft. RS: Investigation, Methodology, Writing – original draft, Writing – review & editing. LL: Project administration, Resources, Writing – original draft. RC: Formal analysis, Funding acquisition, Investigation, Methodology, Resources, Writing – original draft, Writing – review & editing. BA: Conceptualization, Data curation, Formal analysis, Investigation, Methodology, Software, Supervision, Validation, Visualization, Writing – original draft, Writing – review & editing.
