# Supplementary material for: Influence of dietary pattern on anti-tuberculosis treatment outcomes in persons with dysglycemia: a Peruvian prospective cohort study
Source: Front Nutr. 2023 Dec 18;10:1254983. doi: 10.3389/fnut.2023.1254983 (PMC10757910; doi:10.3389/fnut.2023.1254983)
Supplement: Supplementary file 1 [file Data_Sheet_1.pdf]

Supplementary Material

1 Supplementary Tables

Supplementary Table 1. Definitions of tuberculosis treatment outcomes

Outcome	Definition
**Cured**	A pulmonary TB patient with bacteriologically confirmed TB at the beginning of treatment who was smear- or culture-negative in the last month of treatment and on at least one previous occasion
**Treatment completed**	A TB patient who completed treatment without evidence of failure *but* with no record to show that sputum smear or culture results in the last month of treatment and on at least one previous occasion were negative, either because tests were not done or because results are unavailable
**Treatment failed**	A TB patient whose sputum smear or culture is positive at month 5 or later during treatment
**Died**	A TB patient who dies for any reason before starting or during the course of treatment
**Lost to follow-up**	A TB patient who did not start treatment or whose treatment was interrupted for 2 consecutive months or more
**Recurrence/relapse**	Patient who presents another episode of TB diagnosed after being discharged as cured or as treatment completed.
**Not evaluated**	A TB patient for whom no treatment outcome is assigned. This includes cases “transferred out” to another treatment unit as well as cases for whom the treatment outcome is unknown to the reporting unit.
**Treatment success**	The sum of *cured* and *treatment completed*

Table note: Peruvian National TB Program. Definitions and reporting framework for tuberculosis [1].

Supplementary Table 2. Food groups according to similarity in nutritional composition, consumed of the study population.

Food or Food Group	Food Items
**Rice and cereals**	Bread (white or whole), rice (white or whole), noodles (white or whole), flour, oats, green corn or couscous of corn, popcorn salted, homemade cake, biscuit (salted or sweet), pasta soup, *picarones*[a], wheat, quinoa.
**Tubers**	Cassava, sweet potato, potato, yucca, *ullucus*[b].
**Milk and dairy**	Whole milk powder or liquid, skimmed milk powder or liquid, yogurt (whole, diet or light), chocolate ready, yellow cheese, white cheese.
**Fruits and Vegetables**	Lettuce, cabbage, pumpkin, squash, carrot, tomato, chayote, gherkin, beet, okra, vegetable salad, pineapple, avocado, silver banana, ground banana, cashew, papaya, mango, apple, watermelon, melon, orange, tangerine, strawberry, fruit juice or fruit pulp, lettuce, cucumber.
**Legumes**	Beans, lentils, chickpeas, *pallares*[c].
**Meat**	Bovine (fried or cooked), chicken with or without skin (fried or cooked), cooked or fried fish, seafood, viscera, chicken egg (fried or cooked), dehydrated meat (Jerky beef).
**Fast Food**	Fried potatoes, potato chips, pizza, lasagna, ketchup, ready-made soups, sandwich, industrialized salty snack, instant noodles, ready-to-eat sauce and pizza-ready sauce, salty potato.
**Sweetened beverages**	Normal, diet or light soda, artificial juice, carbonated drinks, artificial refreshment, tea, coffee, energy drink and liquid or powdered sweetener.
**Sugar and sweets**	Sugar, chocolate powder, homemade sweets, industrialized sweets, stuffed biscuit, candies, chewing gum, lollipops, chocolate bar, gelatin, ice cream and popsicle (cream and/or chocolate) and *flan*[d].
**Oils**	Butter and vegetable oil.

Table Note: [a]*Picarones*: Peruvian dessert based in squash and sweet potato. [b]Ullucus: a plant grown primarily as a root vegetable, secondarily as a leaf vegetable. [c]*Pallares*: Peruvian beans. [d]*Flan*: is a dessert made-up of eggs and milk with a soft caramel on top.

Supplementary Table 3. Characteristics and relative abundance food group consumption of the study population according to TB treatment outcome

Parameters	Unfavorable n=28	Favorable n=92	p-value
**Age (years), median (IQR)**	49 (30.4-64.9)	27.7 (27.7-39.8)	<0.001
**Male, n. (%)**	19 (67.9)	58 (63)	0.642
**Illiteracy, n. (%)**	7 (25)	25 (27.2)	1
**Prior TB, n. (%)**	5 (17.9)	16 (17.4)	1
**Smoking, n. (%)**	9 (32.1)	16 (17.6)	0.115
**Passive smoking, n. (%)**	2 (7.1)	7 (7.7)	1
**Cannabis use, n. (%)**	5 (17.9)	13 (14.3)	0.763
**Illicit drug use, n. (%)**	4 (14.3)	10 (11)	0.738
**Alcohol use, n. (%)**	16 (57.1)	44 (48.4)	0.518
**Anemia, n. (%)**	22 (78.6)	65 (70.7)	0.477
**BMI (kg/m^2), median (IQR)**	23.9 (21.4-26.4)	22.3 (22.3-24.7)	0.079
**Hb (g/dL), median (IQR)**	11.6 (10.3-13)	12.4 (12.4-13.4)	0.163
**HbA1c (%), median (IQR)**	5.3 (5-6.5)	5.1 (5.1-5.3)	**0.004**
**FPG (g/dL), median (IQR)**	100 (93.2-202.8)	93.5 (93.5-99.9)	**0.003**
**Dysglycemic status**			**<0.001**
**TB-Dysglycemic**	24 (85.7)	28 (30.4)	
**TB**	4 (14.3)	64 (69.6)	
**Relative abundance food group (g/day)**			
**Rice and cereals, median (IQR)**	638.5 (563-650.6)	561.8 (561.8-576.7)	**<0.001**
**Tubers, median (IQR)**	270.7 (233.3-277.7)	233.3 (233.3-233.3)	**<0.001**
**Milk and dairy, median (IQR)**	61.3 (34.7-106.7)	34.7 (34.7-106.7)	0.199
**Fruits and Vegetables, median (IQR)**	13 (13-17.3)	13 (13-17.3)	0.460
**Legumes, median (IQR)**	15.8 (10.5-17.5)	17.5 (17.5-21)	0.251
**Meat, median (IQR)**	41.3 (29.1-49)	34.8 (34.8-43.6)	0.318
**Fast Food, median (IQR)**	150.7 (98.7-426.7)	62.7 (62.7-185.7)	**<0.001**
**Sweetened beverages, median (IQR)**	216.7 (75-266.7)	33.3 (33.3-133.3)	**<0.001**
**Sugar and sweets, median (IQR)**	40 (20-45)	20 (20-32.5)	**<0.001**
**Oils, median (IQR)**	9.3 (5.3-17.3)	4 (4-12)	**0.009**

Table Note: The data presented in continuous variables (represented median and interquartile range [IQR]) between clinical groups were compared using the Mann Whitney test. Qualitative variables were represented by number and frequency (%) and compared using the Pearson's chi-square test.

Abbreviatures: TB: tuberculosis; BMI: body mass index; Hb: Hemoglobin; HbA1c: glycated hemoglobin.

.

Supplementary Table 4. Characteristics and relative abundance of food group consumption of the study population according to Cluster I and Cluster II

Parameter	Cluster I n=51	Cluster II n=69	p-value
**Age (years), median (IQR)**	37.9 (27-51.5)	26.5 (21.7-35.8)	**<0.001**
**Male, n. (%)**	37 (73)	40 (58)	0.124
**Illiteracy, n. (%)**	42 (82.4)	46 (66.7)	0.063
**Prior TB, n. (%)**	12 (23.5)	9 (13)	0.152
**Smoking, n. (%)**	13 (25.5)	12 (17.6)	0.365
**Passive smoking, n. (%)**	4 (7.8)	5 (7.4)	1
**Cannabis use, n. (%)**	10 (19.6)	8 (11.8)	0.303
**Illicit drug use, n. (%)**	10 (19.6)	4 (5.9)	**0.041**
**Alcohol use, n. (%)**	30 (58.8)	30 (44.1)	0.139
**Anemia, n. (%)**	37 (73)	49 (71)	0.836
**Hb (g/dL), median (IQR)**	11.8 (10.3-13.2)	12.4 (11.2-13.3)	0.078
**HbA1c (%), median (IQR)**	5 (5-7)	5 (5-5)	**<0.001**
**FPG (g/dL), median (IQR)**	100.8 (95.3-135.6)	91.3 (85.7-95.9)	**<0.001**
**BMI (kg/m^2), median (IQR)**	23.17 (21.02-25.67)	22.42 (20.4-24.57)	0.42
**Dysglycemic status**			**<0.001**
**TB-Dysglycemic**	44 (86.3)	8 (11.6)	
**TB**	7 (13.7)	61 (88.4)	
**Relative abundance by food group (%)**			
**Sugar and sweets, median (IQR)**	133.33 (66.7-266.7)	33.33 (8.3-133.3)	**<0.001**
**Sweetened beverages, median (IQR)**	40 (20-45)	10 (10-20)	**<0.001**
**Oils, median (IQR)**	13.33 (5.3-21.3)	2.67 (2.7-5.3)	**<0.001**
**Fruits and Vegetables, median (IQR)**	13 (13-17.3)	8.67 (8.7-17.3)	**0.015**
**Legumes, median (IQR)**	17.5 (10.5-17.5)	17.5 (10.5-21)	0.137
**Tubers, median (IQR)**	270.67 (233.3-277.7)	233.33 (163.3-233.3)	**<0.001**
**Fast Food, median (IQR)**	168 (74.7-426.7)	56 (18.7-98.7)	**<0.001**
**Meat, median (IQR)**	41.5 (28.7-57)	33 (25-40.3)	**0.009**
**Milk and dairy, median (IQR)**	80 (42.7-170.7)	34.67 (34.7-61.3)	**0.001**
**Rice and cereals, median (IQR)**	640.07 (566.5-651.2)	558.73 (429.9-565.7)	**<0.001**

Table Note: The data presented in continuous variables (represented median and interquartile range [IQR]) between clinical groups were compared using the Mann Whitney test. Qualitative variables were represented by number and frequency (%) and compared using the Pearson's chi-square test.

Abbreviatures: TB: tuberculosis; BMI: body mass index; Hb: Hemoglobin; HbA1c: glycated hemoglobin.

Supplementary Table 5. Selected main effects in Lasso regression analysis.

	Mean	Standard Deviation	Standardized Coefficients
**Intercept**			0.232
**Sex**	0.62	0.48	0.00
**Positive culture**	0.45	0.50	0.00
**Dysglycemia status**	0.43	0.50	**0.08**
**Prior TB**	0.20	0.40	0.00
**Smoking**	0.23	0.42	0.00
**Dietary Pattern**	0.39	0.49	**0.05**
**Cannabis use**	0.12	0.32	0.00
**Illicit drug use**	0.10	0.30	0.00
**Alcohol consumption**	0.48	0.50	0.00
**Age**	36.58	16.60	**0.02**
**Hemoglobin**	11.98	1.96	0.00
**BMI**	23.37	3.60	0.00

Table Note: Lasso regression analysis was performed using alpha =1. Letters in bold indicate the variables that entered the binary regression model.

Abbreviatures: TB: tuberculosis; BMI: body mass index

2 Supplementary Figures

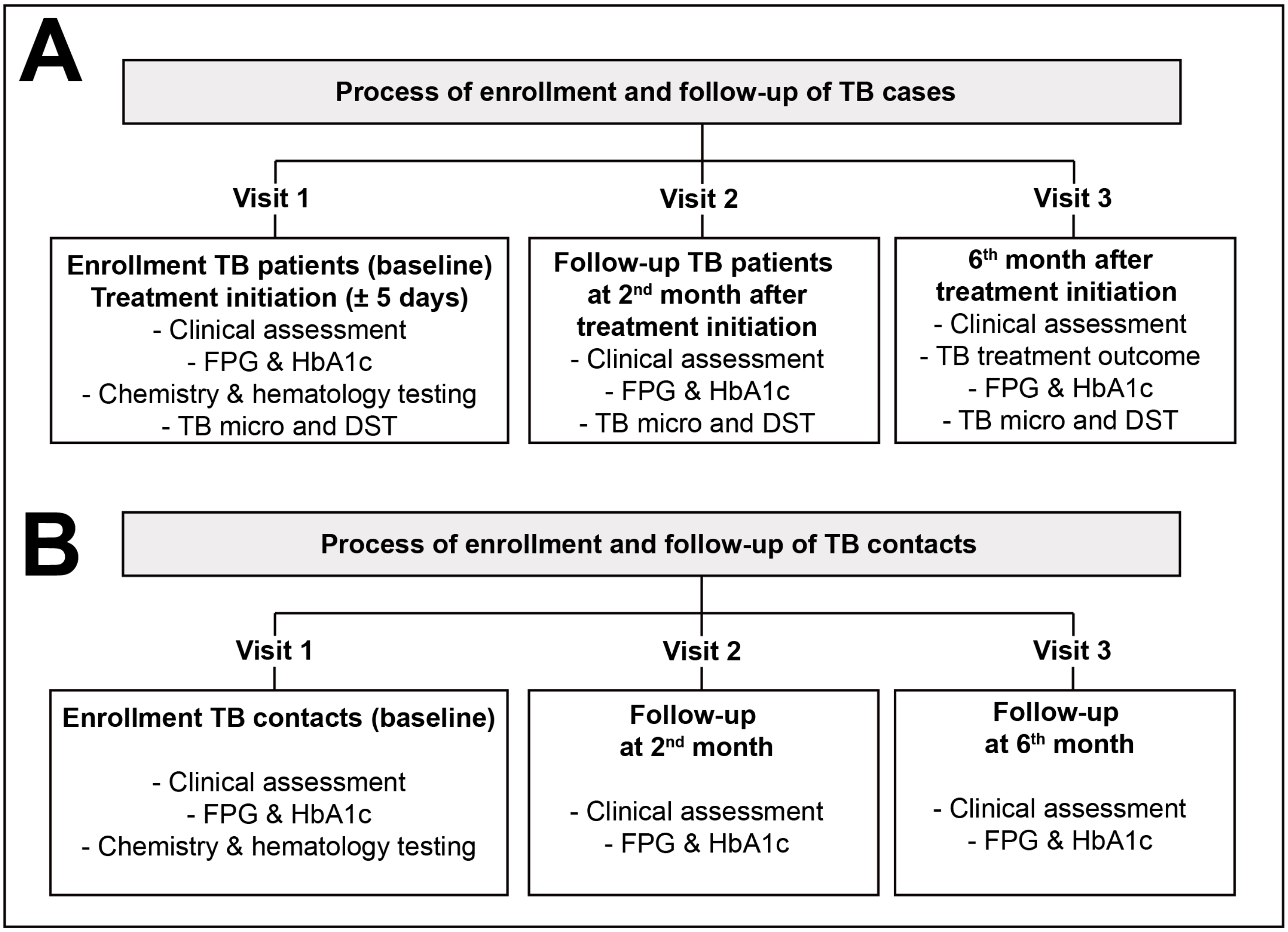

Supplementary Figure 1. Study outline. (A) Flowchart describing the recruitment of the study population. (**B)** Diagram of performed procedures in the baseline and during follow-up. Abbreviatures: TB: tuberculosis; FPG: Fasting Plasma Glucose; HbA1c: Glycated Hemoglobin; DST: Drug susceptibility testing.

References

1. Ministerio de Salud- Perú. Norma técnica de salud para la atención integral de las personas afectadas por tuberculosis, NTS N°104-MINSA. **2013**, *2019*.
